# Improving inferences from short-term ecological studies with Bayesian hierarchical modeling: white-headed woodpeckers in managed forests

**DOI:** 10.1002/ece3.1618

**Published:** 2015-07-22

**Authors:** Daniel W Linden, Gary J Roloff

**Affiliations:** Department of Fisheries & Wildlife, Michigan State UniversityEast Lansing, Michigan, 48824

**Keywords:** Cavity nester, dynamic multistate occupancy, forest management, Gibbs variable selection, nesting probability, *Picoides albolarvatus*, snag retention

## Abstract

Pilot studies are often used to design short-term research projects and long-term ecological monitoring programs, but data are sometimes discarded when they do not match the eventual survey design. Bayesian hierarchical modeling provides a convenient framework for integrating multiple data sources while explicitly separating sample variation into observation and ecological state processes. Such an approach can better estimate state uncertainty and improve inferences from short-term studies in dynamic systems. We used a dynamic multistate occupancy model to estimate the probabilities of occurrence and nesting for white-headed woodpeckers *Picoides albolarvatus* in recent harvest units within managed forests of northern California, USA. Our objectives were to examine how occupancy states and state transitions were related to forest management practices, and how the probabilities changed over time. Using Gibbs variable selection, we made inferences using multiple model structures and generated model-averaged estimates. Probabilities of white-headed woodpecker occurrence and nesting were high in 2009 and 2010, and the probability that nesting persisted at a site was positively related to the snag density in harvest units. Prior-year nesting resulted in higher probabilities of subsequent occurrence and nesting. We demonstrate the benefit of forest management practices that increase the density of retained snags in harvest units for providing white-headed woodpecker nesting habitat. While including an additional year of data from our pilot study did not drastically alter management recommendations, it changed the interpretation of the mechanism behind the observed dynamics. Bayesian hierarchical modeling has the potential to maximize the utility of studies based on small sample sizes while fully accounting for measurement error and both estimation and model uncertainty, thereby improving the ability of observational data to inform conservation and management strategies.

## Introduction

Observational data typical of many ecological studies are often limited in temporal scope, unless the observations are part of a long-term monitoring program. While the quality of inferences from long-term monitoring is unmatched (Lindenmayer and Likens 2009), such efforts are necessarily costly and time-consuming, preventing their application to many conservation and management decisions. In any case, making sound inferences on ecological variables of interest requires that the proper types and amounts of information be collected (Yoccoz et al. 2001). Pilot studies can be used to obtain exploratory information that guides research or monitoring designs (Bailey et al. 2007), but pilot data are often collected in ways that do not match the eventual design and frequently discarded during subsequent analysis (Morris et al. 2013). The information lost from discarding pilot data may be negligible for a long-term study, while for a short-term study, the additional temporal context could prove valuable.

Bayesian hierarchical modeling provides opportunities to conveniently incorporate pilot data and potentially improve inferences from short-term studies. Pilot data can be the basis for informative priors (Morris et al. 2013) or can contribute directly to model likelihoods through integrated approaches that combine information from difference sources (Schaub et al. 2007; Schaub and Kery 2012). Using an explicit hierarchical representation, sample variation can be separated into the ecological processes of interest and the observational processes that result in measurement error, allowing different types of information to contribute directly to each component (Royle and Dorazio 2008). In this way, changes in the observational process can be modeled to accommodate any differences in sampling design. Additionally, using a Bayesian approach to estimation, one can obtain exact inferences regardless of sample size (Fieberg et al. 2013), generate finite-sample estimates (Royle and Kéry 2007), and easily make inferences over multiple models (Tenan et al. 2014). Together, these techniques can improve model accuracy and precision (Morris et al. 2013) and better estimate uncertainty in ecological states, maximizing the information gained from short-term studies.

Natural resource managers often rely on short-term studies to quantify the effectiveness of their management activities (Bennett and Adams 2004). Intensive forest management can result in a nearly complete removal of resources associated with late-successional forests (Franklin 1989), raising concerns about the numerous wildlife species that rely on such resources to meet life history requirements (Thomas et al. 1979; Neitro et al. 1985). In response, forest managers have incorporated measures to mitigate the loss of old-forest attributes in attempts to address biodiversity conservation (Bunnell et al. 1999; Linden et al. 2012). Dead trees, or snags, are ephemeral resources that are particularly difficult to manage for because they are naturally created by stochastic events and eventually decay over time (Morrison and Raphael 1993). In addition to retaining existing snags during timber harvest, some managers have artificially created snags (Kroll et al. 2012). For these measures to effectively conserve viable populations of forest species (Newton 1994), they need to provide environmental conditions suitable for native species at relevant spatial and temporal scales.

During a previous study examining relationships between bird community occupancy and forest management practices on private lands, we observed white-headed woodpeckers *Picoides albolarvatus* more frequently than predicted by regional indices (Linden and Roloff 2013). White-headed woodpeckers were historically associated with old pine forests containing large diameter trees and an open canopy, but this species has been “poorly studied” compared to other woodpeckers in North America (Garrett et al. 1996). More recent research suggests that forest disturbances (e.g., wildfire) that increase snag density and decrease live tree cover can improve habitat quality for white-headed woodpeckers (Wightman et al. 2010; Hollenbeck et al. 2011; Kozma 2011). An understanding of relationships between intensive forest management and white-headed woodpecker ecology may have implications for biodiversity conservation. Primary cavity-nesters often have a disproportionately large influence on the diversity and abundance of bird species by serving as ecosystem engineers (Jones et al. 1994), and some woodpeckers are thought of as keystone species in forest ecosystems (Bednarz et al. 2004; Martin et al. 2004). Forest management practices that address the habitat needs of woodpeckers therefore have the potential to affect entire species assemblages (Daily et al. 1993; Drever and Martin 2010).

Here, we use observations from a short-term study that combined a pilot survey with an expanded second year survey to quantify nesting dynamics of white-headed woodpeckers in managed forests of northern California, USA. We constructed a dynamic, multistate occupancy model (MacKenzie et al. 2009) to (1) estimate probabilities of white-headed woodpecker occurrence and nesting in young clear-cuts, accounting for uncertainty in observations, (2) quantify relationships between nesting and habitat features that are typically modified during timber harvest, and (3) examine changes in these relationships over time. Our hierarchical model estimated parameters using a Bayesian framework to accommodate the small sample sizes, missing data, and unbalanced design. We also used Bayesian model selection procedures to simultaneously fit multiple model structures, quantify evidence of relationships, and generate model-averaged predictions to better incorporate uncertainty. We illustrate how a single additional year of observations can affect inferences about an ecological system, and, more specifically, provide evidence that retained snag density can influence the short-term persistence of white-headed woodpecker nesting in recently harvested forests.

## Methods

### Study area and field protocol

We conducted the study across a ∼12,000 ha portion of private industrial timberlands surrounded by the Shasta–Trinity National Forest, located east of Mt. Shasta in northern California, USA (centered near 41°21′N, 121°50′W). The area was covered by second- and third-growth, mixed-conifer forest that was historically dominated by ponderosa pine (*Pinus ponderosa*) and is now mostly white fir (*Abies concolor*). Selective logging and fire suppression in the last century have altered the overstory composition similar to other dry forests of western North America (Hessburg et al. 2005). True fir (*Abies spp*.) dominance generally increased with elevation, and elevations ranged from 1250 to 1950 m. State laws have limited the amount and juxtaposition of timber harvesting, so landowners often maximize the efficiency of operations by harvesting in a regular pattern across the landscape at 5–10+ year cycles. As a result, the region was characterized by a mosaic of forest stands (i.e., cohorts of trees with similar sizes): ∼41% was mature stands with large diameter trees (>60 cm), ∼36% contained younger stands with medium diameter trees (25–60 cm), and the remaining ∼23% was evenly split among stands with poletimber (12–25 cm), those with saplings (3–12 cm), and recent harvests planted with seedlings (U.S. Forest Service 2007).

**Figure 1:**
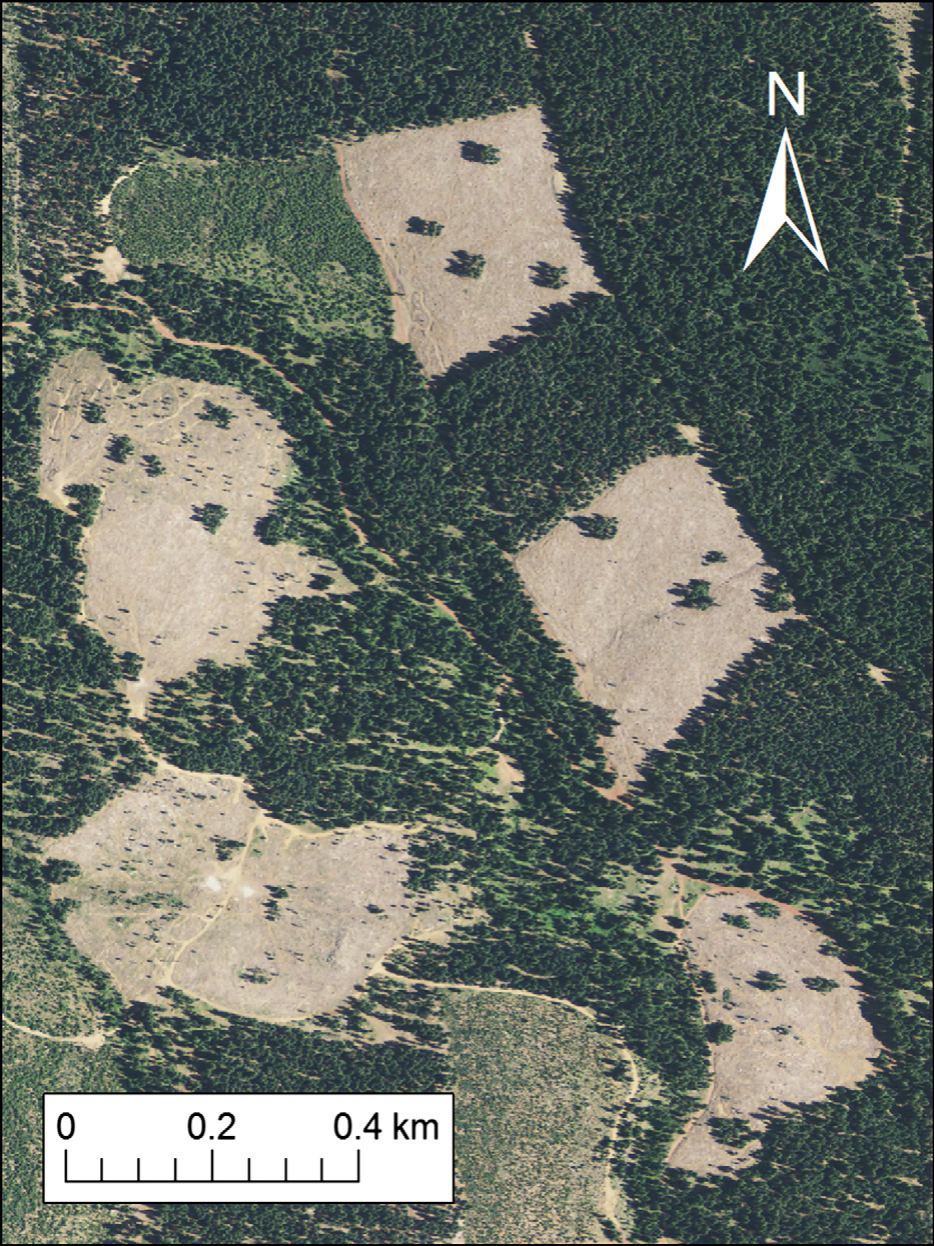
Aerial photograph from 2009 illustrating 5 recent harvest units selected for surveying white-headed woodpecker occurrence and reproduction near Mt. Shasta, California, USA. Several older harvest units (>10 years since harvest) are also visible, along with surrounding mature forest stands. Photograph obtained from National Agricultural Imagery Program (NAIP; http://datagateway.nrcs.usda.gov/). Location centered at 121°51′22″W, 41°22′07″N.

We selected for sampling all recently harvested stands (hereafter, harvest units) in the study area that had been clear-cut during the last major harvesting cycle in 2005 (*n* = 66). Harvest units were identified in a GIS using 2009 aerial photography from the National Agricultural Imagery Program (NAIP; http://datagateway.nrcs.usda.gov/) and maps of timber harvest plans as provided by the California Department of Forestry (http://ftp://ftp.fire.ca.gov/forest/). Harvest units averaged 9 ha (range = 4–15 ha) and typically contained several randomly dispersed patches (∼0.1 ha) of green trees (Fig. 1), in addition to varying densities of snags that were retained during harvest. We measured snag density at each harvest unit with a complete tally of all dead wood >25 cm diameter and >1 m tall. Previous observations suggested tall stumps were a frequently used substrate for cavity nests and were tallied with standing snags; the minimum diameter represented a lower limit for white-headed woodpecker cavity nests observed in previous studies (Milne and Hejl 1989; Kozma 2009).

We surveyed harvest units for white-headed woodpecker presence and nesting during the breeding season (May–July) in 2009 and 2010. The pilot study in 2009 consisted of a single survey where the observer recorded all visual and auditory detections of woodpeckers from a central vantage point in the harvest unit. We used passive as opposed to broadcast surveys (Dudley and Saab 2003) to prevent attracting individuals from nearby harvest units into the focal harvest unit. Surveys lasted <30 min, although majority of initial woodpecker detections occurred <10 min from start time. At the conclusion of the passive survey, areas of the harvest unit where woodpecker behavior suggested the presence of a nest (e.g., individuals viewed carrying food or entering a snag cavity) were searched and observed until a nest was located. All surveys were conducted between sunrise and 5 h post under optimal weather conditions (e.g., no rain or heavy wind). In 2010, we modified the survey design by limiting passive surveys to 10 min and repeatedly visiting harvest units up to 4 times until a nest was discovered. Intervals between the repeated visits were 7–10 days.

We used a removal design (Farnsworth et al. 2002) to construct a detection history of observed states for each harvest unit (1 = undetected; 2 = adult detected; 3 = nest detected), where visits to a given site were terminated once evidence of reproduction was found. Although the detection history for a harvest unit did not contain information after evidence of nesting was found, we continued monitoring cavity nests for other purposes (Linden 2011).

### Occupancy modeling

We constructed a dynamic multistate occupancy model using a hierarchical state-space framework (Royle and Kéry 2007) with a parameterization outlined in MacKenzie et al. (2009) for modeling occupancy with multiple states across multiple seasons. Here, we considered each harvest unit to represent one of *i* = 1, 2,…, *N* = 66 sites having *j* = 1, 2,…, *J* = 4 surveys over *t* = 1, 2 years. The latent true state, *z*_*it*_, at site *i* in year *t* was a partially observed random variable that could be in 1 of 3 states (*m*): unoccupied (*m* = 1); occupied without nesting (*m* = 2); and occupied with nesting (*m* = 3). The true state was considered static within a given year. The observed state, y_*ijt*_, at site *i* during survey *j* in year *t* could be recorded as 1 of 3 states (*l*), conditional on the latent true states. We assumed that state misclassification during observation could only occur in one direction (MacKenzie et al. 2009), such that sites observed to have been unoccupied (*l* = 1) could in reality have been in any 1 of the 3 possible true states, while no uncertainty in state assignment existed for sites observed as occupied with nesting: Pr(*m* = 3|*l* = 3) = 1. We modeled observations conditional on the latent true state such that


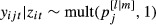
(1)

where 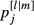 is the probability of the observed state (*y* = *l*) given the true state (*z* = *m*), and because the observed states are mutually exclusive, 
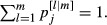
 We used a reparameterization (MacKenzie et al. 2009) where the multinomial probabilities are converted into conditional binomials such that 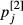 = Pr(*y* = 2*|z* = 2), the probability of observing occupancy given that the site was occupied without nesting; 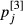 = Pr(*y* = 2*|z* = 3), the probability of observing occupancy given that the site was occupied with nesting; and *δ*_*j*_ = Pr(*y* = 3*|z* = 3), the probability of observing a nest given that the site was occupied with nesting. Under this formulation, the probability of observing a state higher than the true state is a structural zero: Pr(*y* = *l* > *m*|*z* = *m*) = 0. The relationships between the multinomial and binomial probabilities are further illustrated in Appendix S1.

We modeled the latent true occupancy states as random variables from multinomial distributions that were structured dependent on the year. For year 1, the true states were specified such that


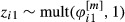
(2)

where 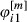 is the probability of state *m* at site *i* in year 1. Similar to the detection probabilities, the state probabilities 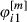 are also mutually exclusive and must sum to 1. We again used a reparameterization converting the multinomial probabilities to conditional binomial probabilities for a more natural ecological interpretation of the parameters (Nichols et al. 2007). As such, we defined *ψ*_*i*1_ = 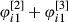 = Pr(*z*_*i*1_ = 2) + Pr(*z*_*i*1_ = 3), the probability that a site is occupied (with or without nesting); and *R*_*i*1_ = 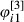/*ψ*_*i*1_ = Pr(*z*_*i*1_ = 3)/(Pr(*z*_*i*1_ = 2) + Pr(*z*_*i*1_ = 3)), the conditional probability of successful reproduction (i.e., nesting) given that a site is occupied. The state probability vector was thus ***ϕ***_*i*1_ = [1 – *ψ*_*i*1_, *ψ*_*i*1_ × (1 – *R*_*i*1_), *ψ*_*i*1_ × *R*_*i*1_]. This formulation allowed for the modeling of covariates that affect *ψ*_*i*1_ and *R*_*i*1_, which was more relevant than, for example, examining relationships with the unconditional probability of occupancy without nesting 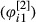.

For year 2, we specified the state probabilities conditional on the true states in year 1 to allow for examination of occupancy state dynamics over time (MacKenzie et al. 2009). As such, the true states in year 2 were modeled as follows:


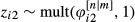
(3)

where 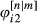 is the probability of state *n* in year 2 given state *m* in year 1, or Pr(*z*_*i*2_ = *n|z*_*i*1_ = *m*). In place of the vector from year 1, the state probabilities in year 2 are now specified according to a 3 × 3 transition probability matrix, ***ϕ***_*i*2_, with each row summing to 1. The reparameterization for year 2 specifies the state transitions ***ϕ***_*i*2_ in terms of *ψ*_*i*2_ and *R*_*i*2_, each of which is conditional on the true state in the previous year, resulting in 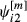 and 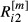 for all *m*. As an example, 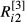 is the probability that a site with nesting in year 1 persists with nesting in year 2. For a more complete description of the probability matrices, see MacKenzie et al. (2009).

We modeled the influence of covariates on the detection and occupancy state probabilities using logit link functions. We considered that detection probability would vary across the breeding season, as individuals became more or less active during mating and offspring rearing activities. For this reason, we included ordinal date as a covariate in each of the logit-linear models of detection:


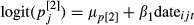
(4)


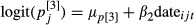
(5)


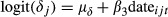
(6)

where the *μ*_*p*[2]_, *μ*_*p*[3]_, *μ*_δ_ are mean detection probabilities on the logit scale and *β*_1_, *β*_2_, and *β*_3_ are regression coefficents for the effect of ordinal date at site *i* during survey *j* in year *t*. Note that we did not allow detection probabilities to vary by year, as the pilot study did not have the repeated site visits necessary to estimate detection. Thus, we assumed the detection process was similar across years and borrowed information from the repeated survey design in year 2 to inform detection in year 1. We subtracted ordinal dates in 2010 by 8 days to accommodate an observed delay in nesting due to abnormal weather, although model results were not sensitive to this.

We limited the modeling of covariates on state probabilities to the process that was of greatest interest to our objectives – the probability of nesting. Initial fitting of the dynamic multistate model indicated that data were too sparse for informing some of the transition probabilities, so we restricted our inferences to the probability of nesting in year 1 (*R*_*i*1_) and the probability of nest persistence 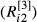, that is, nesting in year 2 given nesting in year 1. We hypothesized that nest availability, as approximated by snag density, would influence the observed variation in nesting across harvest units:


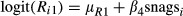
(7)


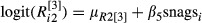
(8)

where *μ*_*R*1_ and *μ*_*R*2[3]_ are mean conditional probabilities of nesting (on the logit scale) for all sites in year 1, and sites in year 2 that had nesting in year 1, respectively; and *β*_4_ and *β*_5_ are regression coefficients for the effect of snag density. All continuous covariates were standardized to a zero mean and unit variance, and snag density was first log-transformed.

### Model selection and parameter estimation

We specified the model using the BUGS language (Lunn et al. 2000) to estimate the joint distribution of the parameters and latent states with Markov chain Monte Carlo (MCMC) methods. We used Gibbs variable selection (GVS), a form of Bayesian model selection (Ntzoufras 2002), to explore multiple model structures and generate model-averaged estimates. Specifically, we used an indicator variable approach to select which of the *β*_*k*_ coefficients corresponding to covariates in the logit-linear models were supported by the data, while simultaneously estimating the coefficient values (Tenan et al. 2014). For each *β*_*k*_ coefficient in *k* = 1,…, *K* = 5, we defined a corresponding indicator *γ*_*k*_ ∼ Bernoulli(0.5), such that the combined linear predictor *γ*_*k*_*β*_*k*_ was either *β*_*k*_ or 0 depending on whether the model included (*γ*_*k*_ = 1) or excluded (*γ*_*k*_ = 0) the matching coefficient for a given MCMC sample. The posterior mean of each *γ*_*k*_ then represented the posterior inclusion probability for the corresponding *β*_*k*_ – how often the model included the regression coefficient during estimation. The prior probabilities for the *β*_*k*_ were specified as a mixture of normal distributions:


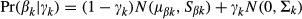
(9)

where *μ*_*βk*_ and *S*_*βk*_ are the mean and variance, respectively, estimated from the posterior of the *β*_*k*_ in a previous model fit using the full structure (i.e., a global model); and Σ_*k*_ is a fixed prior variance. This conditional prior specification does not affect the posterior of *β*_*k*_ but serves to improve mixing of the MCMC sampler (Ntzoufras 2002).

The choice of Σ_*k*_, in contrast, can have a considerable effect on posterior inclusion probabilities and, thus, inferences about the importance of a given covariate (Link and Barker 2006; Tenan et al. 2014). We therefore fit models with different values for Σ_*k*_ ranging from 0.1 to 10,000 at regular intervals on the common log scale (increments of 0.25). This allowed us to examine how the posterior inclusion probabilities, and to some extent the *β*_*k*_ estimates, varied with an increasingly vague prior, recognizing that small variances are actually semi-informative (Tenan et al. 2014). While examining this range of priors provides an informative sensitivity analysis, it would be difficult to draw conclusions and management recommendations from multiple sets upon sets of models. We ultimately chose to make inferences using a prior for Σ_*k*_ proposed by Link and Barker (2006), where the total variance V in the linear predictors is considered to be the sum of the prior variances for the *β*_*k*_ coefficients. The prior for Σ_*k*_ was thus specified as V/(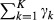 + 11), with the summation over *γ*_*k*_ representing the number of *β*_*k*_ included for a given MCMC sample, added to the fixed number of *μ* intercepts, and V assigned a vague inverse Gamma prior, 1/V ∼ Γ(3.2890, 7.8014). This particular inverse Gamma distribution ensures the marginal prior distributions for the *μ* intercepts are uniform on the unit interval, while still conveying sufficiently vague prior information for the parameters (Link and Barker 2006). In this way, Σ_*k*_ is allowed to vary according to a distribution instead of being specified as a single value chosen a priori. Priors for each of the logit-scale intercepts *μ* were specified as uniform U(0,1) on the probability scale.

The posterior distributions for model parameters represented conditional estimates for the *β*_*k*_ regression coefficients and marginal (model-averaged) estimates for all other parameters. This meant that each *β*_*k*_ posterior distribution was conditional on a model structure that included a contribution to the likelihood from the given *β*_*k*_, occurring when *γ*_*k*_ = 1. For all other parameters (e.g., the *μ* intercepts and latent true states *z*_*it*_), the posterior distributions were model-averaged estimates that were not conditional on a particular model structure, thus incorporating both estimation and model uncertainty. In addition to calculating posterior inclusion probabilities, we calculated model probabilities (sensu Ntzoufras 2002) by assigning a model structure (*M*) to each MCMC sample according to the *γ*_*k*_ indicators: *M* = 1 + 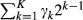. Thus, each model probability, Pr(*M*), represented how often each of the 2^*k*^ = 32 potential model structures was selected during estimation. The final derived parameter that we calculated was a finite-sample estimate using the latent states (Royle and Kéry 2007), representing the proportion of the *N* = 66 sites predicted to have nesting in each year:


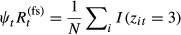
(10)

where *I*(•) is an indicator function. The finite-sample estimates represent realizations of the random variables that will be more precise and potentially more relevant to small sample inferences than the population averages (Royle and Kéry 2007).

We implemented the multistate model using MCMC methods in JAGS (Plummer 2003), with the jagsUI package (Kellner 2014) in R (R Core Team 2015). Our JAGS code is included in Appendix S1. The MCMC sample consisted of 3 chains of 55,000 iterations with a 5000 iteration burn-in period and thinning which resulted in 10,000 values forming the posterior distribution for each model parameter. We determined model convergence by examining trace plots and ensured that the scale reduction factor, or Gelman–Rubin statistic, was <1.1 (Gelman et al. 2013). We present estimates of probabilities, regression coefficients, and other derived parameters with 95% credible intervals and use the posterior inclusion and model probabilities, and effect sizes, to assess the importance of covariates.

## Results

**Figure 2:**
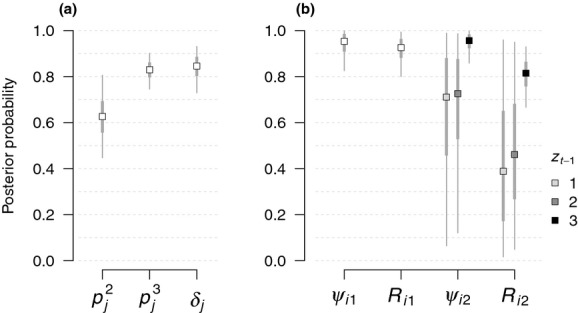
Model-averaged estimates (with 50% and 95% credible intervals) of detection (a) and occupancy (b) probabilities from the dynamic multistate occupancy model for white-headed woodpeckers on private industrial forests in northern California 2009–2010. Detection probabilities include that of an adult given occupancy without nesting 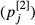, an adult given occupancy with nesting 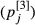, and nesting (*δ*_*j*_). Occupancy probabilities are for adults (*ψ*_*it*_) and nesting given occupancy (*R*_*it*_). The state probabilities for year 2 are conditional on the state in year 1 (*z*_*t*–1_).

White-headed woodpeckers were nesting in most harvest units during the 2 years of observation and were easily observable with high detection probabilities for adults and nests when nesting was present (Fig. 2). The finite-sample proportion of harvest units with nesting, 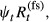 declined from 0.89 (95% CI = 0.79, 0.98) in 2009 to 0.71 (0.67, 0.79) in 2010 (Table 1), with an average difference of 0.17 (0.06, 0.27) between the years. The probabilities of occupancy with or without nesting in 2010 were greater for harvest units with nesting in 2009 than for those without (Fig. 2).

**Table 1:** Posterior means and 95% credible intervals from the dynamic multistate model estimating white-headed woodpecker occupancy and nesting probabilities during 2009–2010 at harvest units in northern California, USA. Parameters include intercepts and regression coefficients from the logit-linear models for probabilities of occupancy (*ψ*_*it*_), nesting given occupancy (*R*_*it*_), detection of an adult given occupancy without nesting 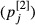, detection of an adult given occupancy with nesting 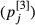, and detection of nesting (*δ*_*j*_). The *μ* intercepts are model-averaged estimates, and those for year 2 are conditional on the state in year 1. The regression coefficient estimates are conditional on model inclusion (*γ*_*k*_ = 1) and represent effects of ordinal date (*β*_1,_ *β*_2_, *β*_3_) on the 3 detection probabilities and snag density (*β*_4_, *β*_5_) on initial nesting probability (*R*_*i*1_) and nesting persistence 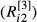. Model-averaged estimates of finite-sample nesting probability 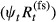 also included

Parameter	Mean	95% CI
*μ*_*ψ*1_	3.27	(1.57, 6.34)
*μ*_*R*1_	2.66	(1.37, 4.84)
*μ*_*ψ*2[1]_	0.87	(–2.65, 4.41)
*μ*_*ψ*2[2]_	1.01	(–1.88, 4.28)
*μ*_*ψ*2[3]_	3.27	(1.84, 5.79)
*μ*_*R*2[1]_	–0.47	(–4.05, 3.03)
*μ*_*R*2[2]_	–0.12	(–3.02, 2.97)
*μ*_*R*2[3]_	1.52	(0.67, 2.53)
*μ*_*p*[2]_	0.54	(–0.22, 1.44)
*μ*_*p*[3]_	1.59	(1.08, 2.19)
*μ*_*δ*_	1.73	(0.99, 2.57)
*β*_1_	0.08	(–0.55, 0.72)
*β*_2_	0.31	(–0.27, 0.90)
*β*_3_	1.96	(1.13, 2.91)
*β*_4_	0.65	(–1.31, 2.54)
*β*_5_	1.74	(0.11, 3.46)
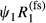	0.89	(0.79, 0.98)
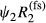	0.71	(0.67, 0.79)

**Figure 3:**
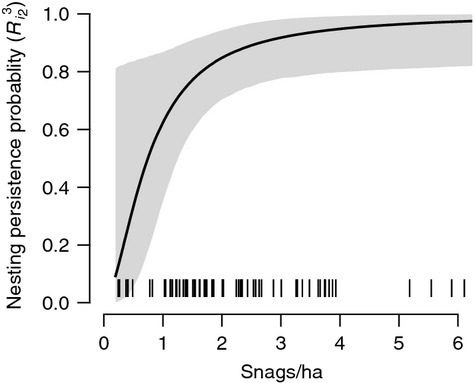
Predicted relationship (mean with 95% credible interval) between white-headed woodpecker nesting persistence 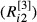 and snag density of harvest units in northern California in 2010, based on conditional estimates of *β*_5_. Observed values indicated by vertical dashes.

**Figure 4:**
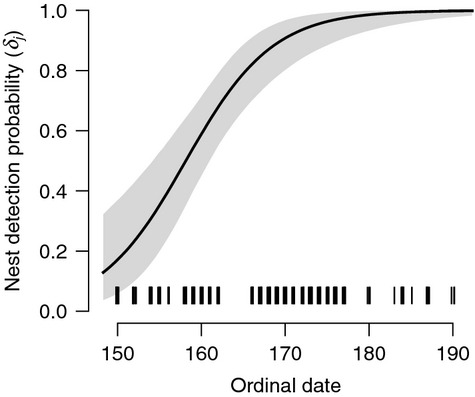
Predicted relationship (mean with 95% credible interval) between probability of detecting a white-headed woodpecker nest (*δ*_*j*_) and ordinal date of survey in northern California in 2010, based on conditional estimates of *β*_3_. Observed values indicated by vertical dashes.

**Figure 5:**
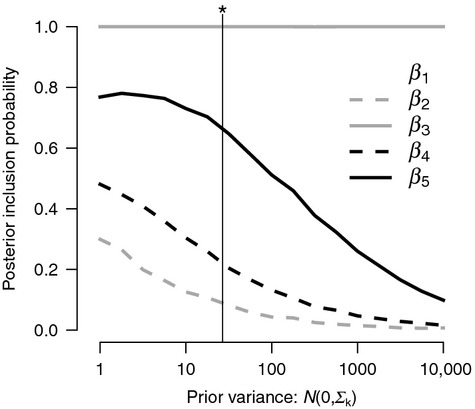
Effects of the prior variance (Σ_*k*_) specified for each *β*_*k*_ regression coefficient and the resulting posterior inclusion probability, Pr(*γ*_*k*_ = 1). The regression coefficients represent effects of ordinal date (*β*_1_, *β*_2_, *β*_3_) on the 3 detection probabilities and snag density (*β*_4_, *β*_5_) on initial nesting probability (*R*_*i*1_) and nesting persistence 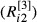. Median posterior value for Σ_*k*_ from the Link and Barker (2006) prior indicated with vertical line (*).

There was strong evidence that nest persistence probability 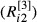 increased with snag density (*β*_5_; Fig. 3) and that detection of a nest (*δ*_*j*_) increased with the ordinal date of the survey (*β*_3_; Fig. 4), as each coefficient had a 95% credible interval that did not overlap zero (Table 1). There was weak evidence that initial nesting probability (*R*_*i*1_) increased with snag density (*β*_4_) and that detection of an adult individual given nesting 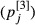 increased with ordinal date (*β*_2_), although the posterior distributions for these coefficients overlapped zero (Table 1). Posterior inclusion probabilities also indicated that *β*_3_ and *β*_5_ were important, and the model structure with the highest posterior probability (0.44) included both coefficients (Table 2), while other coefficients were not as well-supported by the data. The Link and Barker (2006) prior for Σ_*k*_ provided a reasonable compromise between vague and informative values (Fig. 5) while also allowing for uncertainty; the posterior median for Σ_*k*_ under this specification was 26.77 (11.47, 84.61), and *β*_*k*_ estimates were largely shrunken at the lowest variances and had lower posterior inclusion probabilities at higher variances (Appendix S2).

**Table 2:** Posterior probabilities of parameter inclusion, Pr(*γ*_*k*_ = 1), for the *β*_*k*_ regression coefficients, and resulting model structure, Pr(*M*), for the 32 model combinations under the prior specification described in Link and Barker (2006). The regression coefficients represent effects of ordinal date (*β*_1_, *β*_2_, *β*_3_) on the 3 detection probabilities and snag density (*β*_4_, *β*_5_) on initial nesting probability (*R*_*i*1_) and nesting persistence 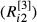. The top model combinations having a cumulative posterior probability <0.95 are displayed, with *γ*_*k*_ indicating which of the *β*_*k*_ coefficients are included for a particular model combination

		γ_*k*_|M						
Parameter	Pr(*γ*_*k*_ = 1)	*M*_21_	*M*_5_	*M*_29_	*M*_13_	*M*_23_	*M*_22_	*M*_7_
*β*_1_	0.06	0	0	0	0	0	1	0
*β*_2_	0.08	0	0	0	0	1	0	1
*β*_3_	1.00	1	1	1	1	1	1	1
*β*_4_	0.21	0	0	1	1	0	0	0
*β*_5_	0.64	1	0	1	0	1	1	0
	Pr(*M*)	0.44	0.24	0.10	0.07	0.04	0.03	0.02

## Discussion

Although white-headed woodpecker occurrence and nesting were prevalent across recent harvest units in northern California, our dynamic multistate occupancy model indicated that the proportion of harvest units with nesting declined by ∼18% from 2009 to 2010. Additionally, the probability of nest persistence at a site (i.e., continued nesting in year 2, given nesting in year 1) increased with snag density in the harvest unit (Fig. 3). These findings suggest that nest-site availability may be a limiting factor for white-headed woodpeckers in this system, a common scenario observed for cavity-nesting species in managed forests (Newton 1994). An initial analysis of these data came to a somewhat similar conclusion regarding the importance of snag density (Linden 2011), yet this previous effort only considered observations from the single year (2010) that used a repeated survey design. Using a hierarchical modeling framework made the inclusion of the 2009 single-visit data trivial, as the separation of the state and observation processes allowed us to use information from the observation process in 2010 to inform observations from 2009 while fully accounting for uncertainty in the ecological states. Without the first year of data, the decline in nesting would not have been apparent, nor would the evidence that nest persistence probability, 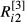, had a much stronger association with snag density than initial nesting probability, *R*_*i*1_. This additional context suggests that nest-site availability may not have been initially limiting postharvest, especially given that ∼90% of harvest units were predicted to have nests in 2009. It is possible that nest-site availability might have become more limiting with time as snags retained during harvest decayed and fell (Russell et al. 2006), although such conclusions must be tempered by the short 2-year period of observation (e.g., no information on a continued trend in 2011 or beyond).

Other studies on white-headed woodpeckers have suggested that decay condition may be more important than density of snags (Raphael and White 1984; Wightman et al. 2010). White-headed woodpeckers are a relatively weak primary cavity excavator, and thus, not all snags are considered available for nest sites when the condition of the wood is unsuitable for cavity excavation (Bagne et al. 2008). Snag decay condition increases with the time-since-death of the tree but also interacts with cause of death (e.g., fire vs. insect) and tree species (Morrison and Raphael 1993). We did not incorporate snag decay condition or tree species in our modeling as the sampled harvest units lacked variation in these attributes: For snags >25 cm diameter, 97% were in moderate to late stages of decay and 86% were true fir (*Abies* spp.). These conditions reflect the similar time-since-harvest (4–5 years) and forest vegetation composition for the sampled sites. Thus, we considered snag density an appropriate proxy for nest-site availability and chose the 25 cm diameter threshold to reflect white-headed woodpecker nest-site preferences demonstrated by previous studies (Milne and Hejl 1989) and our own observations (95% of nest trees >25 cm [*n* = 89]; Linden 2011). We assumed the loss of standing snags between 2009 and 2010 was minimal (Russell et al. 2006), so that our tallies were generally representative of snag density in both years. We also note that while snag density may not have changed between years, other attributes could have influenced the effective nest-site availability for white-headed woodpeckers between years, including interference competition from secondary cavity-nesters (e.g., Northern Flicker *Colaptes auratus*) using old cavities, which was frequently observed (D. Linden, unpublished data). This could explain the importance of increased snag density to nest persistence probability, despite our assumption that snag density was static during the study.

One important assumption of occupancy modeling is that the occupancy state for a given site remains constant over the sampling period, allowing the repeated surveys to provide information about the observation process conditional on the true state (MacKenzie et al. 2002). For multistate occupancy models, this means that a site cannot shift among the multiple possible states (MacKenzie et al. 2009). It is important to consider this assumption in the context of the study objectives and the ecology of the system being observed. In our study, changes in state might happen for a site with an early or late-season nest failure, where the observed state of occupied with nesting changes to simply occupied or even unoccupied (if the site is completely abandoned). It could also happen when a site is occupied by an adult that does not eventually nest (e.g., unsuccessful mate pairing) and leaves the area. While early season nest failures may have gone unobserved, we had ancillary information on the status of monitored nests during each season and observed high nest survival rates (>0.85; Linden 2011). We also modeled the change in detectability as nests progressed from eggs to fledglings to account for an increased ability to observe nests later in the season (Fig. 4). We chose not to remove late-season failed nests as the incidence was low and cause of failure was typically predation, unrelated to factors affecting nest availability. Thus, our inferences relate to an “effective” state for each site and our estimates of nesting probability were mostly proportional to the probability of successful nesting (Martin et al. 2009).

In using a Bayesian approach to estimation, our modeling effort benefited in several ways. Bayesian inference provides a coherent solution to uncertainty without having to rely on the asymptotic assumptions of maximum likelihood estimators, an advantage most relevant to small sample sizes (Royle and Dorazio 2008; Fieberg et al. 2013). The data demand for a dynamic multistate occupancy model can be large, as the state transition probability matrix ***ϕ*** requires an adequate collection of sites transitioning between the various states in order to estimate all probabilities (MacKenzie et al. 2009). While our estimates for certain state transitions were poor due to low sample sizes, the priors constrained the estimates to a reasonable parameter space and the uncertainty was fully propagated throughout the model, allowing us to make inferences on the important transitions that could be adequately estimated (e.g., 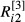). We could have formally tested whether certain transitions were significantly different and pooled those that were not (Martin et al. 2009), but decided to focus our inferences on how transitions related to nesting were affected by habitat. Using GVS for model selection (Ntzoufras 2002), we were able to simultaneously examine the importance of several covariates and derive model-averaged estimates of the latent states and their probabilities. Despite uncertainty caused by the selection of a prior variance for the *β*_*k*_ regression coefficients (Fig. 5), our use of the Link and Barker (2006) prior enabled us to make inferences that balanced model complexity and parsimony. These findings demonstrate the utility of such a prior specification to applications of Bayesian model selection.

Our results suggest that forest management practices which incorporate the retention of snags during harvest are beneficial to white-headed woodpecker nesting across industrial forests of northern California, at least in the short term. Harvest units with a snag density >2 snags·ha^−1^ (stems >25 cm diameter) had a nest persistence probability of 0.85 (0.70, 0.94); almost half of the harvest units surveyed in 2009–2010 had a snag density above this level. State forest practice rules in California require the retention of all nonmerchantable safe snags during harvest (California Department of Forestry and Fire Protection 2007). It is likely that initial snag densities were higher when harvesting occurred in 2005 than when harvest units were surveyed in 2010 given average rates of snag longevity (Russell et al. 2006), although the many factors that influence snag dynamics make such an assessment difficult (Morrison and Raphael 1993). Snags that were created during harvest (e.g., tall stumps, broken stems) may have taken 3–5 years before even being suitable for cavity excavation (Arnett et al. 2010); thus, nest-site availability may actually have peaked during our woodpecker surveys. Longer monitoring would be necessary to quantify the extent to which our observed decline in nesting probability continues over time, although fewer snags and increased vegetative cover for predators would reduce the nesting habitat quality for white-headed woodpeckers (Wightman et al. 2010). At the landscape scale, the continued aging of harvest units will coincide with the approach of the next harvesting cycle, which would potentially provide new nesting opportunities in adjacent harvest units. With additional data, our dynamic multistate occupancy model could elicit more complex metapopulation dynamics (Sutherland et al. 2014) by examining changes in transition probabilities over time with factors such as age-since-harvest and juxtaposition of old and new harvest units. This information would be useful to forest managers seeking to balance wood extraction goals with wildlife conservation, particularly as it pertains to management decisions at stand and landscape scales (Lindenmayer and Franklin 2002).

## Data Accessibility

JAGS code: uploaded as online supporting information. Occupancy survey data: uploaded as online supporting information.

## Supporting Information

**Appendix S1.** JAGS code and data used to fit the dynamic multistate model.

**Appendix S2.** Posterior inclusion probabilities, means, and standard errors of *β*_*k*_ regression coefficients with increasing prior variance Σ_*k*_.
