# Supplementary material for: Improving inferences from short-term ecological studies with Bayesian hierarchical modeling: white-headed woodpeckers in managed forests
Source: Ecol Evol. 2015 Jul 22;5(16):3378–88. doi: 10.1002/ece3.1618 (PMC4569033; doi:10.1002/ece3.1618)
Supplement: Supplementary file 1 — Appendix S1. JAGS code and data used to fit the dynamic multistate model. [file ece30005-3378-sd1.doc]

**Appendix S1.** JAGS code and data used to fit the dynamic multistate model. The code is annotated to provide general descriptions of model components. The model specifications are consistent with the formatting in JAGS which requires that normal distributions be specified in terms of precision (inverse variance) instead of standard deviation; precision is indicated by τ. Greek symbols from model specifications in the text are typically spelled out in the model code (e.g., ψ = psi, μ = mu). Index letters in the code are consistent with those in the text, though ordering is sometimes changed for looping purposes. The names of all data types (vectors, arrays) required by the model are indicated at the start and provided at the end.

# Data:

# N = # sites

# T = # years

# J[i,t] = # surveys for site i in year t

# y[i,j,t] = observed state (l) at site i, survey j, year t

# date[i,j,t] = ordinal date of survey j at site i in year t

# snags[i] = snag density at site i

# mu_beta_ps[k] = mean for beta k from global model where g[k]~dbern(1)

# tau_beta_ps[k] = tau for beta k from global model where g[k]~dbern(1)

model{

# state m=1: unoccupied; state m=2: occupied;, state m=3; occupied w/ nest

for (i in 1:N){

#logit occupancy model for m=2 in year 1

logit(psi_[i,1]) <- mu_psi1

psi[i,1] <- max(0.001,min(0.999, psi_[i,1]))

#logit occupancy model for m=3 in year 1

logit(R_[i,1]) <- mu_R1 + g[4]*beta[4]*snags[i]

R[i,1] <- max(0.001,min(0.999, R_[i,1]))

Phi0[i,1] <- 1-psi[i,1] # Pr(z=1)

Phi0[i,2] <- psi[i,1]*(1-R[i,1]) # Pr(z=2)

Phi0[i,3] <- psi[i,1]*R[i,1] # Pr(z=3)

# true state in year 1

z[i,1] ~ dcat(Phi0[i,])

for (t in 2:T){

#logit occupancy model for m=2 in year t given m in year t-1

logit(psi_[i,t]) <- mu_psi[z[i,t-1]]

psi[i,t] <- max(0.001,min(0.999, psi_[i,t]))

#logit occupancy model for m=3 in year t given m in year t-1

logit(R_[i,t]) <- mu_R[z[i,t-1]] +

equals(z[i,t-1],3)*g[5]*beta[5]*snags[i]

R[i,t] <- max(0.001,min(0.999, R_[i,t]))

# Note number ordering swapped from text: t,t+1 instead of t|t-1

# Also, Phi needs a t index for analyses with T>2

# Pr(z[t+1]=1|z[t]=m)

Phi[i,1,1] <- 1-psi[i,t]

Phi[i,2,1] <- 1-psi[i,t]

Phi[i,3,1] <- 1-psi[i,t]

# Pr(z[t+1]=2|z[t]=m)

Phi[i,1,2] <- psi[i,t]*(1-R[i,t])

Phi[i,2,2] <- psi[i,t]*(1-R[i,t])

Phi[i,3,2] <- psi[i,t]*(1-R[i,t])

# Pr(z[t+1]=3|z[t]=m)

Phi[i,1,3] <- psi[i,t]*R[i,t]

Phi[i,2,3] <- psi[i,t]*R[i,t]

Phi[i,3,3] <- psi[i,t]*R[i,t]

# true m in year t, given t-1

z[i,t] ~ dcat(Phi[i,z[i,t-1],])

}

}

for (i in 1:N){

for (t in 1:T) {

for (j in 1:J[i,t]) {

#p[site,survey,year,z(m),y(l)]

p[i,j,t,1,1] <- 1 # Pr(y=1|z=1)

p[i,j,t,1,2] <- 0 # Pr(y=2|z=1)

p[i,j,t,1,3] <- 0 # Pr(y=3|z=1)

p[i,j,t,2,1] <- 1-p2[i,j,t] # Pr(y=1|z=2)

p[i,j,t,2,2] <- p2[i,j,t] # Pr(y=2|z=2)

p[i,j,t,2,3] <- 0 # Pr(y=3|z=2)

p[i,j,t,3,1] <- 1-p3[i,j,t] # Pr(y=1|z=3)

p[i,j,t,3,2] <- p3[i,j,t]*(1-delta[i,j,t]) # Pr(y=2|z=3)

p[i,j,t,3,3] <- p3[i,j,t]*(delta[i,j,t]) # Pr(y=3|z=3)

# linear trend for detection (ordinal date)

logit(p2[i,j,t]) <- mu_p2 + g[1]*beta[1]*date[i,j,t]

logit(p3[i,j,t]) <- mu_p3 + g[2]*beta[2]*date[i,j,t]

logit(delta[i,j,t]) <- mu_delta + g[3]*beta[3]*date[i,j,t]

y[i,j,t] ~ dcat(p[i,j,t,z[i,t],])

}}}

#Priors for probabilities

psi1_prob ~ dunif(0,1)

R1_prob ~ dunif(0,1)

p2_prob ~ dunif(0,1)

p3_prob ~ dunif(0,1)

delta_prob ~ dunif(0,1)

mu_psi1 <- log(psi1_prob/(1-psi1_prob))

mu_R1 <- log(R1_prob/(1-R1_prob))

mu_p2 <- log(p2_prob/(1-p2_prob))

mu_p3 <- log(p3_prob/(1-p3_prob))

mu_delta <- log(delta_prob/(1-delta_prob))

for(m in 1:3){

psi_prob[m] ~ dunif(0,1)

R_prob[m] ~ dunif(0,1)

mu_psi[m] <- log(psi_prob[m]/(1-psi_prob[m]))

mu_R[m] <- log(R_prob[m]/(1-R_prob[m]))

}

# prior from Link & Barker (2006) Ecology

tau.V ~ dgamma(3.2890,7.8014)

sumg <- sum(g)+11

tau <- tau.V/sumg

# GVS priors

for(k in 1:5){

beta_mu[k] <- (1-g[k])*mu_beta_ps[k]

beta_tau[k] <- g[k]*tau + (1-g[k])*tau_beta_ps[k]

beta[k] ~ dnorm(beta_mu[k],beta_tau[k])

g[k] ~ dbern(0.5) # set p=1 for global model

}

# Model number for each iteration

mdl <- 1 + g[1]*1 + g[2]*2 + g[3]*4 + g[4]*8 + g[5]*16

for(mod in 1:32){

pmdl[mod] <- equals(mdl,mod)

}

} #end model

jags_data <-

structure(list(N = 66L, T = 2, J = structure(c(1, 1, 1, 1, 1,

1, 1, 1, 1, 1, 1, 1, 1, 2, 1, 1, 1, 1, 1, 1, 1, 1, 1, 1, 1, 1,

1, 1, 1, 1, 1, 2, 1, 1, 1, 1, 1, 1, 1, 1, 1, 1, 1, 1, 1, 1, 1,

1, 1, 1, 1, 1, 1, 1, 1, 1, 1, 1, 1, 1, 1, 1, 1, 1, 1, 1, 3, 2,

3, 2, 3, 1, 2, 1, 2, 3, 1, 1, 2, 2, 2, 3, 2, 2, 1, 2, 1, 2, 2,

3, 1, 3, 2, 1, 2, 2, 2, 1, 3, 1, 2, 1, 2, 3, 2, 1, 3, 2, 2, 2,

2, 4, 3, 2, 2, 2, 2, 2, 2, 2, 2, 2, 1, 2, 2, 2, 1, 1, 2, 1, 1,

1), .Dim = c(66L, 2L)), y = structure(c(2, 3, NA, 2, 1, 3, 2,

3, 1, 3, 3, 2, 3, 2, 3, 3, 3, 2, 3, 3, 3, 3, 3, 3, 3, 3, 3, 2,

3, 1, 3, 1, 3, 3, 3, 3, 3, 3, 1, 3, 3, 3, 1, 3, 1, 3, 3, 1, 1,

3, 3, 3, 1, 3, 3, 1, 3, 3, 1, 1, 3, NA, NA, 1, 3, 3, NA, NA,

NA, NA, NA, NA, NA, NA, NA, NA, NA, NA, NA, 3, NA, NA, NA, NA,

NA, NA, NA, NA, NA, NA, NA, NA, NA, NA, NA, NA, NA, 2, NA, NA,

NA, NA, NA, NA, NA, NA, NA, NA, NA, NA, NA, NA, NA, NA, NA, NA,

NA, NA, NA, NA, NA, NA, NA, NA, NA, NA, NA, NA, NA, NA, NA, NA,

NA, NA, NA, NA, NA, NA, NA, NA, NA, NA, NA, NA, NA, NA, NA, NA,

NA, NA, NA, NA, NA, NA, NA, NA, NA, NA, NA, NA, NA, NA, NA, NA,

NA, NA, NA, NA, NA, NA, NA, NA, NA, NA, NA, NA, NA, NA, NA, NA,

NA, NA, NA, NA, NA, NA, NA, NA, NA, NA, NA, NA, NA, NA, NA, NA,

NA, NA, NA, NA, NA, NA, NA, NA, NA, NA, NA, NA, NA, NA, NA, NA,

NA, NA, NA, NA, NA, NA, NA, NA, NA, NA, NA, NA, NA, NA, NA, NA,

NA, NA, NA, NA, NA, NA, NA, NA, NA, NA, NA, NA, NA, NA, NA, NA,

NA, NA, NA, NA, NA, NA, NA, NA, NA, NA, NA, NA, NA, NA, NA, NA,

NA, NA, NA, NA, 2, 2, 1, 2, 2, 3, 2, 1, 2, 1, 3, 3, 1, 2, 1,

1, 1, 1, 3, 1, 3, 1, 2, 2, 3, 2, 2, 3, 1, 3, 2, 3, 1, 3, 2, 3,

2, 1, 2, 3, 2, 3, 2, 2, 2, 2, 1, 2, 1, 2, 2, 2, 2, 1, 2, 2, 3,

2, 2, 2, 3, 3, 2, 1, 3, 3, 2, 2, 2, 3, 2, NA, 1, NA, 1, 1, NA,

NA, 1, 3, 3, 2, 3, 1, NA, 1, NA, 3, 3, 1, NA, 2, 3, NA, 3, 3,

3, NA, 1, NA, 3, NA, 3, 2, 3, NA, 1, 3, 3, 3, 3, 2, 1, 3, 1,

3, 1, 3, 1, 3, 1, 2, NA, 3, 2, 1, NA, NA, 2, NA, NA, NA, 1, NA,

1, NA, 2, NA, NA, NA, NA, 2, NA, NA, NA, NA, NA, 3, NA, NA, NA,

NA, NA, NA, NA, 2, NA, 3, NA, NA, NA, NA, NA, NA, 1, NA, NA,

NA, NA, 3, NA, NA, 3, NA, NA, NA, NA, 2, 3, NA, NA, NA, NA, NA,

NA, NA, NA, NA, NA, NA, NA, NA, NA, NA, NA, NA, NA, NA, NA, NA,

NA, NA, NA, NA, NA, NA, NA, NA, NA, NA, NA, NA, NA, NA, NA, NA,

NA, NA, NA, NA, NA, NA, NA, NA, NA, NA, NA, NA, NA, NA, NA, NA,

NA, NA, NA, NA, NA, NA, NA, NA, NA, NA, NA, 2, NA, NA, NA, NA,

NA, NA, NA, NA, NA, NA, NA, NA, NA, NA, NA, NA, NA, NA, NA, NA

), .Dim = c(66L, 4L, 2L)), date = structure(c(0.8, 0.8, 0, -0.8,

-0.8, -0.8, -1.3, -0.7, -1.3, -0.7, -0.7, -0.7, 0.6, -1.3, 0.6,

0.6, 0.4, 0.8, 0.7, 0.6, 0.4, -0.7, 0.8, 0.7, 0.7, -0.7, 0.7,

-0.6, 0.3, 0.3, 0.4, 1, 1, -0.6, 0.3, 0.3, 1, 1, 1, 1, 0.4, 0.3,

1, 0.2, 0.2, 0.4, 0.2, 0.2, 1.3, 1.3, 1.3, -0.5, -0.5, -0.5,

-0.5, -0.6, -0.6, -0.6, -0.6, -0.6, -0.6, 0, 0, 0.9, 0.9, 0.3,

NA, NA, NA, NA, NA, NA, NA, NA, NA, NA, NA, NA, NA, 0.8, NA,

NA, NA, NA, NA, NA, NA, NA, NA, NA, NA, NA, NA, NA, NA, NA, NA,

1.6, NA, NA, NA, NA, NA, NA, NA, NA, NA, NA, NA, NA, NA, NA,

NA, NA, NA, NA, NA, NA, NA, NA, NA, NA, NA, NA, NA, NA, NA, NA,

NA, NA, NA, NA, NA, NA, NA, NA, NA, NA, NA, NA, NA, NA, NA, NA,

NA, NA, NA, NA, NA, NA, NA, NA, NA, NA, NA, NA, NA, NA, NA, NA,

NA, NA, NA, NA, NA, NA, NA, NA, NA, NA, NA, NA, NA, NA, NA, NA,

NA, NA, NA, NA, NA, NA, NA, NA, NA, NA, NA, NA, NA, NA, NA, NA,

NA, NA, NA, NA, NA, NA, NA, NA, NA, NA, NA, NA, NA, NA, NA, NA,

NA, NA, NA, NA, NA, NA, NA, NA, NA, NA, NA, NA, NA, NA, NA, NA,

NA, NA, NA, NA, NA, NA, NA, NA, NA, NA, NA, NA, NA, NA, NA, NA,

NA, NA, NA, NA, NA, NA, NA, NA, NA, NA, NA, NA, NA, NA, NA, NA,

NA, NA, NA, NA, NA, NA, NA, NA, -0.9, 0.9, -1.7, -1.1, -1.1,

-0.9, -0.9, -0.9, -0.9, -0.9, -0.9, 0.5, -0.8, -1.7, -1.2, 0,

-0.8, -1.7, 0, -1.2, -0.8, -1.7, -1.2, -1.2, 0, -1.7, 0, 0, 0.5,

-1.2, -1.7, -0.8, -0.8, 0, -1.7, -0.1, -0.8, -0.8, -0.8, -0.8,

-1.7, -0.1, -0.8, -1.5, -1.5, -1.7, -1.5, -1.5, -1.5, -1.5, -1.3,

-1.3, -1.3, -1.3, -1.3, -1.3, 0.2, 0.2, 0.2, -1.3, -1.3, 0.6,

0.6, -1.1, -0.9, -0.1, 0.5, 1.7, 0.5, 0.9, 0.9, NA, 1.7, NA,

1.7, 0.5, NA, NA, 0.8, 0.9, 0.7, 0.8, 0.8, 0.9, NA, 0.8, NA,

0.5, 0.7, 0, NA, -0.1, 0.7, NA, 2.3, -0.1, -0.1, NA, 0.9, NA,

0.6, NA, 0.9, 0.9, 2, NA, -0.1, 2, 0.9, 0.1, 0.1, -0.1, 0.1,

0.1, 0.1, 0.1, 0.1, 0.2, 0.2, 0.2, 0.2, 0.2, NA, 2.3, 2.3, 0.2,

NA, NA, 2, NA, NA, NA, 1.7, NA, 2, NA, 1.7, NA, NA, NA, NA, 1.7,

NA, NA, NA, NA, NA, 1.8, NA, NA, NA, NA, NA, NA, NA, 0.7, NA,

0.6, NA, NA, NA, NA, NA, NA, 2, NA, NA, NA, NA, 2, NA, NA, 0.6,

NA, NA, NA, NA, 0.6, 0.7, NA, NA, NA, NA, NA, NA, NA, NA, NA,

NA, NA, NA, NA, NA, NA, NA, NA, NA, NA, NA, NA, NA, NA, NA, NA,

NA, NA, NA, NA, NA, NA, NA, NA, NA, NA, NA, NA, NA, NA, NA, NA,

NA, NA, NA, NA, NA, NA, NA, NA, NA, NA, NA, NA, NA, NA, NA, NA,

NA, NA, NA, NA, NA, NA, NA, 1.7, NA, NA, NA, NA, NA, NA, NA,

NA, NA, NA, NA, NA, NA, NA, NA, NA, NA, NA, NA, NA), .Dim = c(66L,

4L, 2L)), snags = c(0.27962, -0.31206, -0.51689, -0.41007, -0.53262,

0.13838, 1.15374, -0.35907, 0.24848, 0.54076, -0.26264, 0.0456,

-0.004, 0.49596, -0.756, 0.72969, 0.38452, -0.43612, 0.29177,

-1.89787, 0.7623, 0.26578, -0.21327, -0.01669, 0.77959, 0.75924,

0.65306, -0.81204, 0.05246, 1.08508, -0.35486, -0.23636, 0.28873,

0.1384, 0.42283, -0.02759, 0.73895, 0.62108, 0.14578, -0.1354,

-0.35076, 0.06121, -0.12406, 0.80899, 0.3701, 0.40749, 1.21413,

-0.07487, -1.46606, -0.43761, -1.54514, -0.07496, -1.98141, -0.52103,

-1.27965, 0.32983, 0.62735, -0.22617, -0.44515, -1.46145, -0.07639,

1.24982, -0.07427, -0.1505, 0.79442, 0.68788), mu_beta_ps = structure(c(0.0733, 0.3061, 1.9431, 0.6324, 1.6936), .Dim = 5L), tau_beta_ps = structure(c(9.6117, 10.8106, 4.798, 1.0528, 1.4214), .Dim = 5L)), .Names = c("N", "T", "J", "y", "date", "snags", "mu_beta_ps", "tau_beta_ps"))
